# News and Miscellany

**Published:** 1902-04

**Authors:** 


### News and Miscellany.

**Austin and San Antonio** are rigidly enforcing the anti-spitting law.

---

**The Insane Asylum** at Austin has been equipped with a fire escape in the shape of a spiral "chute," which is the latest thing in fire escapes.

---

**Creosote in Pneumonia:**

℞ Creosot. carbonat ............................ ʒiv

Glycerini............ .......................... ℥i

Aqua Menthæ ............................ ad Oss

M. Dose: Tablespoonful in water.

---

**One on the Doctor.**—"No, I am not a Christian Scientist," said Kendor.

"But you believe in throwing physic to the dogs," remarked Dr. Krabbed.

"Not if it happened to be your physic and my dogs."—*Ex.*

---

**Notice.**—We have a good opportunity for a physician with from $3500 to $4000 who wishes to change from country or smaller town to a larger city. Will bear strictest investigation. For further particulars address "Public Service Exchange," Alamo Insurance Building, San Antonio, Texas.

---

**Remember the Date.**—The great Confederate Reunion will take place at Dallas, 22nd to 25th of this month. The Association of Medical Officers of the Army and Navy of the Confederacy will meet in the judicial room of the city hall in Dallas, Texas, corner of Akard and Commerce streets, on Tuesday, April 22, 1902, at 12 m.

---

**The Governor** has appointed the following named physicians of this State as delegates from Texas to the American congress of tuberculosis, to be held in New York City, on May 14th to 16th, 1902: Dr. W. H. Allen, Marlin; Dr. I. P. Sessions, Rockdale; Dr. M. M. Smith, Austin; Dr. J. M. Inge, Denton; Dr. T. H. Stallcup, Jefferson; Dr. H. A. West, Galveston; Dr. F. Paschal, San Antonio.

**Dr. Bennett** has a locomobile. Splurging down one of the Austin streets the other day—he met Dr. Steiner in his buggy. Dr. Steiner's horse, an old residenter, was so astonished at seeing an Austin doctor cutting such a splurge that he ran away,—tried to "go way back and sit down,"—take a back seat, for he read in the thing the doom of all horse flesh. Dr. Steiner was thrown out, but escaped serious injury.

---

**Texas Not In It.**—Smallpox hardly exists in Japan. This exemption from the disease can be attributed to systematic vaccinaion and revaccination. Careful revaccination has been proved again a complete safeguard against smallpox, as during the past year it is said no case of the disease has occurred among the staff of the London smallpox hospitals. Even poor old Spain has eradicated smallpox in a large measure, by compulsory vaccination. Alas, poor Texas!

---

**The Texas State Medical Association** will meet in Dallas, May 6th to 9th. It is expected that this meeting will be the largest and most important one within the history of the Association. An unusually attractive program has been prepared, and executive business of great importance is to be transacted. Many distinguished physicians from other States are expected to be present, amongst them the great surgeon Murphy, of Chicago, who will, it is said, give us a talk on surgery of the prostate. Lamphear will be there.

---

**Political Influence of Physicians.**—There are enough physcians in every State to control medical legislation if they will only act together. Thackeray says that any woman without an actual hump can marry any man she pleases; man's safety is that woman are like the beasts of the field; they do not know their own strength. The same might be said of the physicians of the country; they have not appriciated their own power. If quackery thrives and gains recognition and privilege under the law it is the fault of an inactive and unorganized medical profession.—*Journal American Medical Association.*

---

**Death of Dr. Ashton.**—Lawrence Ashton, M. D., New York University, 1885, one of the leading physicians of Northern Texas, and a member of the American Medical Association, died at his home in Dallas, March 6, after a brief illness, aged 57. He was a private student of the late Prof. A. L. Loomis, of New York. During his residence in Texas he was an active worker in the State

Medical Association; he was a member of the legislative committee two years ago, and was an earnest advocate of the present law governing the practice of medicine. He was at the time of his death president of the faculty of the Dallas Medical College, medical department of Trinity University and professor of practice of medicine. He was a native of Virginia and a Virginia gentleman.

---

**Creosote and Creosote.**—Merck & Co., in a communication to the American Pharmaceutical Association, call attention to a great danger into which the careless or unwary doctor may fall in prescribing creosote. Only beech wood creosote should be used internally. We reproduce a Merck label in which is contained the caution: "Caution.—Whenever creosote is indicated for internal medication, this kind (wood creosote) should be dispensed; and under no circumstances should so-called "creosote" from coal-tar be given internal use unless explicitly so directed. Wood creosote and "coal-tar creosote" are two different substances. They do not consist of the same chemical ingredients; and they differ very largely in their action on the human body. Wood creosote is comparatively harmless; while "coal-tar creosote" is distinctly poisonous. A substitution of "coal-tar creosote" for wood creosote may, therefore, cause the gravest consequences.

---

**Abortive Treatment of Smallpox.**—The following, from the venerable author of the abortive treatment of smallpox by the free use of sponging with a solution of bichloride of mercury, will be read with interest. Some confusion it seems, was created by the doctor, in his earlier communications, speaking of a "ten per cent. solution":

CLEBURNE, TEXAS, March 9, 1902.

*Editor Texas Medical Journal, Austin, Texas.*

To a letter from Dr. Miller, Pittsburg, Pa., asking for my ten per cent. bichloride of mercury to half a gallon of water, I replied, saying that one grain of the salt saturated twenty-six times its weight of water, and it is the ten per cent. of that solution that was wanted; or sixty-five grains of bichloride to sixty-four ounces of water. Leaving out fractions this is an accurate calculation of the same, and as it had not occurred to me before, that there was a doubt in the mind of some members of the profession as to the proper proportion, I would have been more particular in making the statement long ago. I beg you then, as the starter of the rational treatment of smallpox in your journal, to insert this note in the next issue of the "Red Back," and oblige.

Your old friend,

T. C. OSBORNE, M. D.

